# Ecological Niche and Positive Clusters of Two West Nile Virus Vectors in Ontario, Canada

**DOI:** 10.1007/s10393-023-01653-8

**Published:** 2023-11-20

**Authors:** Benoit Talbot, Manisha A. Kulkarni, Maxime Rioux-Rousseau, Kevin Siebels, Serge Olivier Kotchi, Nicholas H. Ogden, Antoinette Ludwig

**Affiliations:** 1 School of Epidemiology and Public Health, University of Ottawa, Ottawa, ON Canada; 2 Research Group on Epidemiology of Zoonoses and Public Health (GREZOSP), Faculty of Veterinary Medicine, Université de Montréal, Saint-Hyacinthe, QC Canada; 3 Public Health Risk Sciences Division, National Microbiology Laboratory, Infectious Disease Prevention and Control Branch, Public Health Agency of Canada, Saint- Hyacinthe, QC, and Guelph, ON Canada; 4 Signal, Image Processing and Multimedia (STIM), Research Unit and Digital Expertise (UREN), Université Virtuelle de Côte d’Ivoire, Abidjan, Côte d’Ivoire

**Keywords:** *Culex pipiens/restuans*, *Aedes vexans*, West Nile virus, Kulldorf’s spatial scan statistic, Machine learning, Random forest algorithm

## Abstract

West Nile virus (WNV) is a mosquito-borne pathogen associated with uncommon but severe neurological complications in humans, especially among the elderly and immune-compromised. In Northeastern North America, the *Culex pipiens/restuans* complex and *Aedes vexans* are the two principal vector mosquito species/species groups of WNV. Using a 10-year surveillance dataset of WNV vector captures at 118 sites across an area of 40,000 km^2^ in Eastern Ontario, Canada, the ecological niches of *Cx. pipiens/restuans* and *Aedes vexans* were modeled by random forest analysis. Spatiotemporal clusters of WNV-positive mosquito pools were identified using Kulldorf’s spatial scan statistic. The study region encompasses land cover types and climate representative of highly populated Southeastern Canada. We found highest vector habitat suitability in the eastern half of the study area, where temperatures are generally warmer (variable importance > 0.40) and residential and agricultural cropland cover is more prominent (variable importance > 0.25). We found spatiotemporal clusters of high WNV infection rates around the city of Ottawa in both mosquito vector species. These results support the previous literature in the same region and elsewhere suggesting areas surrounding highly populated areas are also high-risk areas for vector-borne zoonoses such as the WNV.

**Supplementary Information:**

The online version contains supplementary material available at 10.1007/s10393-023-01653-8.

## Introduction

First discovered in Uganda in 1937 (Smithburn et al. 1940), West Nile virus (WNV) now has a wide global geographic range, including North America (Gubler 2007; Reisen 2013; Chancey et al. 2015). In North America, including Canada, a range of passerine bird species act as reservoir hosts, and the virus is transmitted among birds via several mosquito vector species (Kilpatrick et al. 2006; Marini et al. 2017). A large number of mammalian, reptilian and amphibian species, including humans, may be incidental hosts of the virus (Artsob et al. 2006). Human cases of infection by WNV (Chancey et al. 2015) are typically asymptomatic and therefore rarely reported (McDonald et al. 2021). However, some infections may develop into neuroinvasive disease, especially among certain risk groups, such as those of older age, and/or those with chronic comorbidities, such as diabetes and hypertension (Badawi et al. 2018).

Several mosquito species have been identified as being particularly efficient at transmitting the virus, because of their intrinsic vector competence, their abundance, their ornithophilic or opportunistic feeding habits and the habitat in which they live, among other factors (Reisen 2013). In North America, mosquito species of the genus *Culex*, such as *Culex pipiens* and *Culex restuans*, are recognized as important vectors maintaining the enzootic cycle of WNV among avian hosts. *Cx. pipiens* and *Cx. restuans* also occasionally feed on mammals, potentially bridging transmission between birds and humans (Ebel et al. 2005; Patrican et al. 2007; Hamer et al. 2008, 2009; Farajollahi et al. 2011; Andreadis 2012). Mosquitoes of the genus *Aedes*, such as *Ae. vexans*, are highly opportunistic in their choice of hosts, meaning they can also effectively bridge transmission between birds and humans (Molaei and Andreadis 2006; Greenberg et al. 2013; Anderson et al. 2020). These species are involved in transmission cycles among avian hosts in Eastern Canada and may bridge transmission to humans from early summer through to fall (Giordano et al. 2018). An association between abundance and minimum infection rates by the WNV in the two species/species groups was observed in the city of Ottawa, Canada, and was predictive of rates of WNV infection in mosquito pools (Talbot et al. 2019b), which suggests more suitable habitats for these mosquitoes may also be hot spots for WNV transmission.

Canada is currently experiencing profound climate change, and this, as well as land use change, is expected to increase the risk of zoonotic diseases, including WNV (Allan et al. 2003; Canadian Paediatric Society 2008; Chen et al. 2013; Kim et al. 2014; Gardner et al. 2014; Hoover and Barker 2016; Ludwig et al. 2019; Rakotoarinia et al. 2022). Indeed population density, landscape, climate and weather have been found to be predictive of human WNV incidence in Ontario, Canada (Giordano et al. 2017; Mallya et al. 2018). Therefore, there is a pressing need for an enhanced understanding of the environmental hazard for WNV on which to base refinements to WNV surveillance and management activities. Spatiotemporal clustering approaches have been used extensively to identify disease transmission hotspots, offering information for targeting surveillance activities (Ansari et al. 2020). Ecological niche models also offer predictive information that may be useful for public health, informing on spatial distributions of habitats most suitable for disease vectors (Peterson 2006; Escobar and Craft 2016; Escobar 2020).

*Cx. pipiens/restuans* demonstrate a higher preference for urban and suburban environments owing to its ability to breed in artificial containers, stagnant pools of ground water and catch basins (McMahon et al. 2008; Gardner et al. 2014; Giordano et al. 2018; Holmes and Cáceres 2020; Cloutier et al. 2021). *Aedes vexans* has a specifically opportunistic nature, thriving in any disturbed humid environment, including anthropogenic ones (Berec et al. 2014; Rocheleau et al. 2017; Giordano et al. 2018; Hopkins et al. 2019; Holmes and Cáceres 2020; Cloutier et al. 2021). Climatic factors have been shown to be predictive of the abundance of these species in Southeastern Canada (Wang et al. 2011; Chuang et al. 2012; Dussault et al. 2018; Ripoche et al. 2019; Rakotoarinia et al. 2022).

In this study, the ecological niche of the two main WNV vector species using data from a 10-year study period in a region of 40,000 km^2^ in Southeastern Canada, in the province of Ontario, was investigated. The presence of WNV-positive mosquito clusters in these data were also explored. Based on the previous literature, we hypothesized that hotspots of WNV incidence would be in areas of higher vector habitat suitability and would be located near the largest urban centers of the study area.

## Materials and Methods

### Study Area and Duration

**Figure 1:**
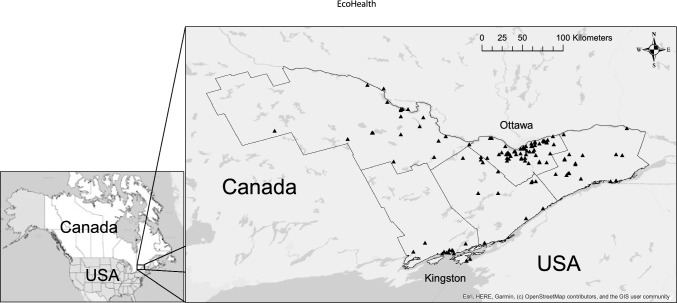
Study area in Eastern Ontario, Canada, with the 118 sampling sites represented by black triangles. The cities of Ottawa and Kingston are shown, and an inset map visually places the study area in North America. Map was created using ArcGIS 10.5 (ESRI, Redlands, CA, USA).

The study area is defined by the following five health units of Ontario, Canada: City of Ottawa; Eastern Ontario; Leeds, Grenville and Lanark; Renfrew; and Kingston, Frontenac and Lennox and Addington, covering a total area of more than 40,000 km^2^. It is predominantly a forested and agricultural landscape with continental humid climate. The two major urban population centers in our study area are Ottawa and Kingston (Fig. 1).

### Mosquito Collection Data

Across this study area, Public Health Ontario (PHO) collects data on mosquito surveillance at sites where a human population is present, which consists of mosquito capture and identification of 22 species/species groups. Capture is performed using light traps designed by the Centers for Disease Control and Prevention (CDC; Atlanta, GA, USA) baited with dry ice for CO_2_ to increase collection effectiveness. Immediately following capture, mosquitoes are stored on dry ice and transported to the laboratory for identification of species of adult female mosquitoes. Arboviral testing of certain species, prioritized by PHO according to arbovirus transmission risk, is then performed to detect WNV; females are pooled in groups of a maximum of 50 individuals of the same species from the same sampling visit, referred to as mosquito pools. *Cx. pipiens/restuans* is prioritized over all other species, and *Ae. vexans* is also of high priority. RNA is extracted from each pool using RNeasy Mini Kit (Qiagen, Hilden, Germany). RNA extracts are tested by quantitative polymerization chain reaction for arbovirus presence (Lanciotti et al. 2000).

### Land Cover and Climatic Data

Land cover data for the year 2016 were obtained from Agriculture and Agri-Food Canada (AAFC) and from the United States Geological Survey (USGS) with a resolution of 30 m across our study area. Data for the year 2016 were chosen because it is approximately the midpoint of our study period from 2011 to 2020. Annual crop inventory data from AAFC (Agriculture and Agri-Food Canada 2022) comprise seven land cover classes: open water, wetlands, agricultural croplands, natural grasslands, forests, exposed surface and residential areas. Residential areas were subdivided into four categories, according to the normalized difference vegetation index (NDVI) from USGS, created using Landsat 8 data (collection 2, level 2, maximum 50% clouds) from May to October 2016, from USGS (Masek et al. 2006; Vermote et al. 2016). The goal of this procedure was to subdivide urban environments according to presence of vegetation, which may affect habitat selection by the studied species. Residential areas with NDVI <  = 0.15 were referred as non-vegetated, with NDVI > 0.15 and <  = 0.30 as low-green, with NDVI > 0.30 and <  = 0.60 as medium-green and with NDVI > 0.60 as high-green residential areas. Data from AAFC and USGS used the same resolution with matching cell frames, and therefore, merging of the two datasets could be performed manually.

While months when mosquitoes are least active, i.e., colder months, are also when temperatures are below the threshold for WNV replication (Dohm and Turell 2001; Dohm et al. 2002; Shocket et al. 2020), temperatures and precipitations in the cold season may affect survival and adult mosquito abundance in the following warm season (Eisen et al. 2014; Roiz et al. 2014; Giménez et al. 2015). Therefore, we considered temperature and precipitation data across the entire study duration. Temperature and precipitation data were obtained from the National Aeronautics and Space Administration (NASA). Daily maximum and minimum temperature and daily total precipitation data were extracted from the daily surface weather and climatological summaries from NASA (Thornton, M.M. et al. 2020) with a resolution of 1,000 m. These data were modified to obtain average of mean daily temperature and average of total daily precipitations for the total period across our study area.

### Ecological Niche Modeling Analysis

The two studied species were observed at least once in the majority of the 118 sampling locations over the study period (at only one site were both species absent and at two sites *Cx. pipiens/restuans* were absent). Therefore, the number of visits in which each species was observed at least once at each site was calculated, and divided by the total number of visits at each site over the study period. The resulting value is the frequency of observed presence and is a form of aggregated performance measure often used in species distribution models (Halvorsen 2012). Sites were then dichotomized into a 0/1 distribution, which is a requirement of the approach: sites with 50% or more species occurrence and sites with less than 50%. This classification informs on habitat selection behavior in the two species, rather than survival thresholds or opportunistic occurrence.

As absence and presence of a mosquito species in our dataset are likely to be affected by the same sampling bias (Phillips et al. 2009; Sillero and Barbosa 2021), the random forest algorithm was used. In general, this decision tree-based approach performs as good as the maximum entropy approach (Beeman et al. 2021; Zhao et al. 2022) and better than traditional regression-based approaches when using large datasets sampled over a long duration and a large spatial scale (Mondal and Bhat 2021). These analyses were performed using the “biomod2” package (Thuiller et al. 2009) in R 4.2.1 (R Development Core Team, Vienna, Austria). All land cover and climatic datasets were projected to Albers Conic Equal Area, which was the original projection of the annual crop inventory dataset. All explanatory variables were resampled to a cell size of 120 × 120 m and set to be at the same cell frame to reduce spatial bias caused by unequal resolution with the mosquito dataset (Sillero and Barbosa 2021). Pearson’s correlation coefficient was calculated among climatic variables at sampling sites, to identify collinearity among variables. Variables were considered collinear if *r* > 0.7. The prevalence parameter was set to 0.5 was specified, meaning “presence” and “absence” distributions are considered in equal proportions in the analysis (Barbet-Massin et al. 2012). For each species, 100 replicate models were trained using 80% of data. To evaluate each model, we computed a receiver operating characteristic (ROC) using the remaining 20% of data. Data was selected randomly in each model for training versus testing. The final model, trained by the 100 replicate models and using 100% of data, was used to generate a habitat suitability index (HSI) map in the study area. All other parameters were kept at default values. Models with ROC above 0.75 were used to generate an ensemble niche model (Thuiller et al. 2009). Response plots of the mean HSI across models and committee averaging (Araujo and New 2007) were generated, for each explanatory variable. Variable importance for each explanatory variable, which varies from 0 to 1, was calculated using a procedure of 100 permutations from the ensemble niche model.

### Spatiotemporal Cluster Detection

Kulldorf’s spatial scan statistic implemented in the SaTScan 9.4 software (Kulldorff 1997) was used to identify possible spatiotemporal clusters with high rates of WNV-positive mosquito pools from 2011 to 2020 across the study area. The analysis was conducted separately for *Cx. pipiens/restuans* and *Ae. vexans*. The Poisson model was used, using the number of mosquitoes of the same species that were tested together as denominator. A likelihood ratio test was performed to identify clusters with significantly higher or lower relative risk than expected, defined as the observed versus expected number of cases. Data aggregation was performed for each week (seven days) of the study, which matches the maximum temporal resolution of our data, and specified 10% of the population at risk, which is recommended by the author when only specific sites in the study area were sampled, as opposed to the whole area (Kulldorff 1997). Other parameters were kept at default values. Spatial and temporal overlap of spatiotemporal clusters of WNV-positive mosquito pools were compared across the study area.

## Results

### Mosquito Collection Data

**Figure 2:**
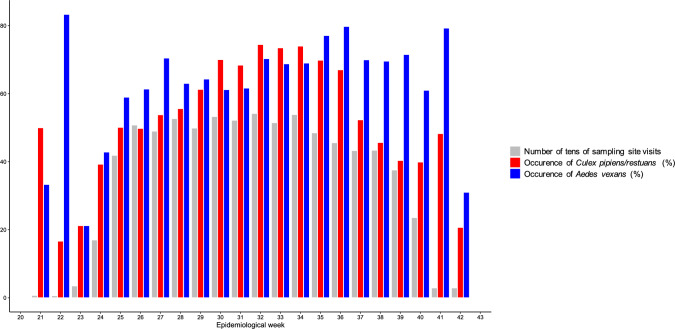
Histogram showing total number of tens sampling visits (number of sampling visits divided by 10) and occurrence (frequency of observed presence) of *Culex pipiens/restuans* and *Aedes vexans* adult mosquitoes, across sampling sites and years, for each epidemiological week.

Sampling sites were visited at least once and at most weekly from June to September each year across the study period. A total of 45 sites (out of 118) were visited 30 times or less across the study period. Sampling sites were visited mostly during the epidemiological weeks in the period when both *Cx. pipiens/restuans* and *Ae. vexans* were most likely to be observed, i.e., weeks 24 to 40 (Fig. 2). Among sites with 30 visits or less across the study period, only one site had a visit (out of 13) outside this timeframe, specifically at week 23 of 2020. We therefore decided to keep all the data in our analysis, considering each visit had a reasonable chance to find one of the targeted species during a visit. A total of more than 110,000 *Ae. vexans* and more than 40,000 *Cx. pipiens/restuans* individuals were collected at an average of 66 sampling visits per site during the study period (Table 1). While close to three times as many *Ae. vexans* than *Cx. pipiens/restuans* individuals were collected, occurrence across sampling visits was similar between the two species (Table 1; Table S1). The number of pools tested for WNV was higher for *Cx. pipiens/restuans* due to higher priority of *Cx. pipiens/restuans* for WNV testing by PHO, and total number of positive pools was also higher for *Cx. pipiens/restuans* (Table 1; Table S1). However, total number of *Cx. pipiens/restuans* individuals tested was lower, which is due to smaller pools for that species on average compared to *Ae. vexans*, and minimum infection rate was much higher for *Cx. pipiens/restuans* (Table 1).

**Table 1:** Descriptive Statistics Relating to the Abundance, Occurrence and West Nile virus (WNV) Testing for Adult Mosquitoes from Both Studied Species, *Culex pipiens/restuans* and *Aedes vexans*, Collected Across 118 Sampling Locations in Eastern Ontario, Canada, 2011–2020.

Species	*Culex pipiens/restuans*	*Aedes vexans*
Total sampled adult mosquitoes	43,807	118,561
Number of visits/site (mean ± standard deviation)	66 ± 57	66 ± 57
Abundance/visit (mean ± standard deviation)	5 ± 7	17 ± 46
Occurrence/visit (mean ± standard deviation)	0.57 ± 0.24	0.65 ± 0.18
Total number of WNV detection tests	4,711	3,617
Number of positive pool [% of total for all species]	179 [95]	8 [4]
Total number of adult mosquitoes tested for West Nile virus	41,294	52,844
Number of adult mosquitoes tested/test (mean ± standard deviation)	9 ± 12	15 ± 16
Total minimum infection rate (positive pools/number tested × 1000)	4.33	0.15

### Land Cover and Climatic Data

**Figure 3:**
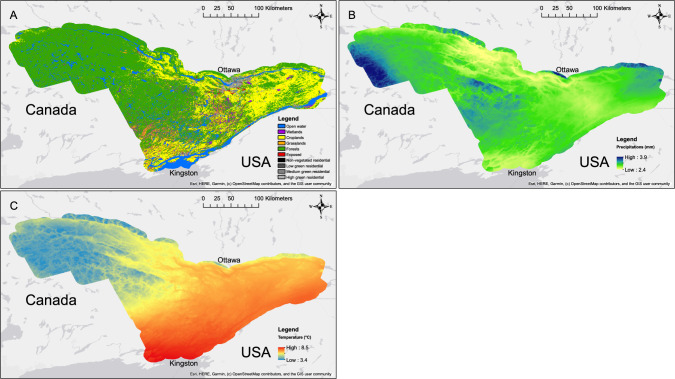
Spatial representation of spatial datasets used in the study. Land cover for the year 2016 **A**, daily total precipitations averaged from 2011 to 2020 **B** and daily mean temperature averaged from 2011 to 2020 **C** are shown. Map was created using ArcGIS 10.5 (ESRI, Redlands, CA, USA).

**Figure 4:**
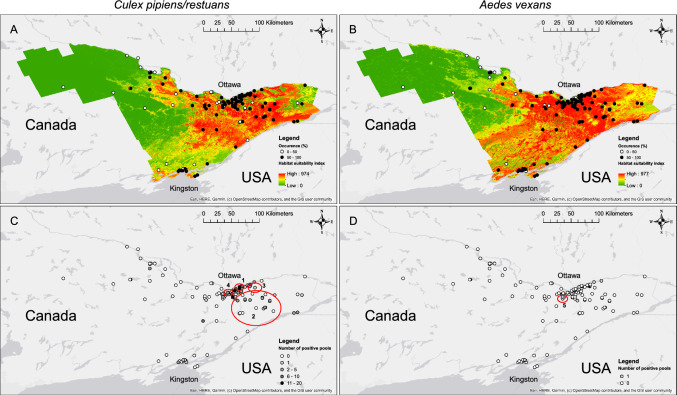
Projected habitat suitability index (**A**–**B**) and significant spatiotemporal clusters of positive mosquito pools (**C**–**D**), and for both study species, *Culex pipiens/restuans* (**A**, **C**) and *Aedes vexans* (**B**, **D**), collected across 118 sampling locations in Eastern Ontario, Canada, 2011–2020. **A**–**B** Sampling locations were sorted into two categories, based on whether they were observed less often than not (occurrence = 0– 50%) or more often than not (occurrence = 50–100%), as in Legend. Habitat suitability index was projected using the best-performing model for each species. **C**–**D** Sampling locations were sorted into categories based on the number of positive pools detected at each one, as in Legend. Spatiotemporal clusters of positive mosquito pools are shown in red circles and identification labels refer to those in Table 3. Map was created using ArcGIS 10.5 (ESRI, Redlands, CA, USA).

Land cover consisted of a total of 10 classes. The two most abundant across the study area are the forested and agricultural cropland classes: the former being characteristic of the western part of the study area and the latter being characteristic of the eastern part (Fig. 3). All residential classes, and wetlands, grasslands and exposed classes were mostly present in the corridor between the cities of Ottawa and Kingston (Fig. 3). The open water class was mostly represented by the Ottawa and Saint Lawrence rivers, which delimit the study area in the north and south, and large waterbodies mainly in the western part of the study area (Fig. 3). Average daily total precipitation varied between 2.3 and 3.8 mm across the study area. The western part of the study area, and some areas along the Ottawa river in the north, received more precipitation on average, while areas northwest and southeast of Ottawa, and north of Kingston received less precipitations on average (Fig. 3). Average daily mean temperature varied between 3.4 and 8.5°C, and the western part of the study area was much colder on average than the eastern part, while the warmest area being the Kingston area in the south (Fig. 3). Open water, wetlands, exposed land, grasslands and low-green residential land cover classes were the least common at sampling sites, being present at fewer than 10 sampling sites (Table 2; Table S1). Habitat suitability indices computed for these land cover classes are therefore of limited use. Wetlands and exposed classes occurred at fewer than three sampling sites (Table 2). The non-vegetated residential class was extremely rare across the study area (Fig. 3) and occurred at no sampling site (Table 2). Therefore, no habitat suitability index could be computed in locations characterized by it (Fig. 4). Average total precipitations and mean temperature at cells containing a sampling site were 2.7 mm and 6.9°C, respectively (Tables 2, 3; Table S1).

**Table 2:** Number of Sampling Sites (% of all sampling sites) Situated in Cells of Each Land Cover Class, Average (standard deviation) Total Precipitations and Mean Temperature at Cells Containing a Sampling Site.

Explanatory variable	Class	Value
Land cover	Open water	6 (5)
	Wetlands	1 (1)
	Croplands	17 (14)
	Grasslands	3 (3)
	Forested	25 (21)
	Exposed	2 (2)
	Non-vegetated residential	0 (0)
	Low-green residential	3 (3)
	Medium-green residential	22 (19)
	High-green residential	39 (33)
Precipitations (mm)		2.70 (0.12)
Temperature (°C)		6.88 (0.66)

**Table 3:** Start and End date, Observed (Obs) and Expected (Exp) number of cases, relative risk (RR), likelihood ratio (LLR) and *P* value of detected space–time clusters of high West Nile virus rates for both study species, *Culex pipiens/restuans* and *Aedes vexans*, collected across 118 sampling locations in Eastern Ontario, Canada, 2011–2020. Cluster identification number (#) refer to those in Fig. 4 C-D.

Species	Start date	End date	Obs	Exp	RR	LLR	*P*	#
*Culex pipiens/restuans*	2015/08/14	2017/09/28	19	3	7	21	< 0.01	1
	2017/08/04	2018/09/06	13	1	11	19	< 0.01	2
	2016/08/26	2017/09/07	8	1	15	14	0.01	3
	2012/07/20	2012/09/06	8	1	12	12	0.03	4
	2012/07/27	2012/09/06	7	1	13	11	0.06	N/A
	2013/09/13	2017/08/24	0	7	0	7	0.82	N/A
*Aedes vexans*	2017/08/25	2017/09/14	2	0	856	11	0.02	5
	2014/09/05	2017/09/07	2	0	12	3	0.92	N/A
	2013/06/14	2017/10/05	0	1	0	1	0.97	N/A
	2016/06/17	2020/12/31	0	1	0	1	1.00	N/A

### Ecological Niche Modeling Analysis

The correlation coefficient between average total daily precipitation and average mean daily temperature was low (*r* = 0.09). Overall, receiver operating characteristic (ROC)’s area under the curve (AUC) values were much higher for *Ae. vexans* models compared to *Cx. pipiens/restuans* models. Out of 100 models, the best performing model had a ROC’s AUC = 0.77 for Cx. *pipiens/restuans* compared to 0.92 for *Ae. vexans* (Table 4). The ensemble model contained 4 and 24 models, respectively for Cx. *pipiens/restuans* and *Ae. vexans*, with a mean ROC’s AUC higher than 0.9 for mean habitat suitability index (HSI) values and committee averaging and for both species (Table 4). Explanatory variable importance of the temperature variable in the ensemble model was higher than 0.4 and higher than the importance of the other two explanatory variables. Importance of land cover and precipitation were both higher than 0.3 for Cx. *pipiens/restuans* and lower than 0.3 for *Ae. vexans* (Table 4).

**Table 4:** Parameters Used to Generate Ensemble Niche Models for Both Studied Species, *Culex pipiens/restuans and Aedes vexans*, Collected Across 118 Sampling Locations in Eastern Ontario, Canada, from 2011 to 2020. Number of Models Included out of the 100 Random Forest Models Generated, Response Operating Curves for the Best-Performing Model, for the Mean Habitat Suitability Index (HSI) Averaged Across Models, and Committee Averaging of HSI Across Models and Variable Importance Calculated with a 100-Permutation Procedure for the Three Explanatory Variables are Shown.

Species		*Culex pipiens/restuans*	*Aedes vexans*
Number of models		4	24
Response operating curve	Best-performing model	0.77	0.92
	Mean HSI values	0.96	0.97
	Committee averaging	0.95	0.96
Variable importance	Land cover	0.34	0.25
	Precipitations	0.32	0.19
	Temperature	0.46	0.44

Projected HSI of the best performing model for both species led to an area of highest HSI value (974 and 977 for *Cx. pipiens/restuans* and *Ae. vexans* respectively, with highest possible maximum value of 1000) concentrated in the eastern part of the study area for both species, centered on and surrounding the city of Ottawa, comprising a wide band around the city, and along the banks of the Ottawa and Saint Lawrence rivers (Fig. 4A-B). The area of highest HSI was broader for *Ae. vexans* than for *Cx. pipiens/restuans* with more suitable areas north of the city of Kingston and in the extreme eastern part of the study area, but mostly areas that were suitable for *Cx. pipiens/restuans* were largely also suitable for *Ae. vexans* (Fig. 4A-B).

**Figure 5:**
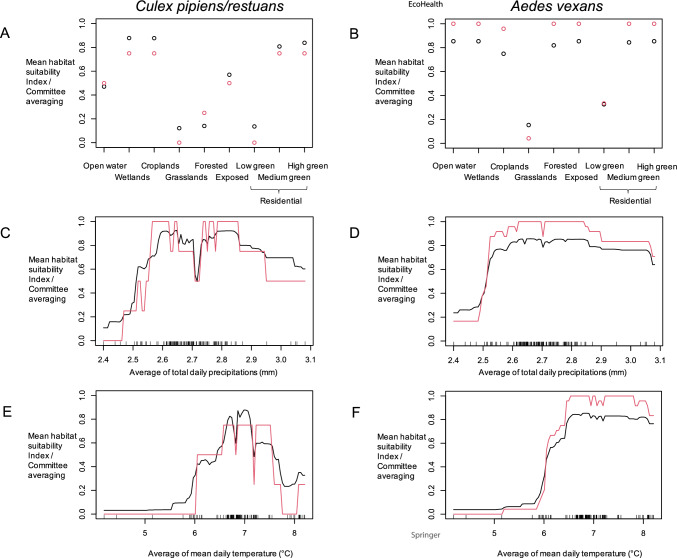
Habitat suitability index calculated for land cover **A**–**B**, total precipitations **C**–**D** and mean temperature **E**–**F** of the ensemble niche model for *Culex pipiens/restuans* **A**, **C**, **E**) and *Aedes vexans* **B**, **D**, **F**. Black and red dots/lines represent mean habitat suitability index and committee averaging, respectively, across models included in the ensemble niche model.

Mean HSI and committee averaging showed high suitability of wetlands, croplands and the two most vegetated residential land cover classes for both species, although open water, forested and exposed classes also showed high suitability for *Ae. vexans* (Fig. 5A-B). Mid-range values of daily total precipitations, i.e., between roughly 2.6 and 2.8 mm, were associated with the highest suitability values, according to both mean HSI and committee averaging, for both species, with low precipitation values were associated with the lowest suitability values (Fig. 5C-D). Higher daily mean temperature values, i.e., above roughly 6°C, were associated with the highest suitability values, according to both mean HSI and committee averaging, for both species, although very high temperature values, i.e., higher than 7°C, were associated with lower suitability than upper-mid-values for *Cx. pipiens/restuans* (Fig. 5E–F).

### Spatiotemporal Cluster Detection

A total six spatiotemporal clusters of positive pools in *Cx. pipiens/restuans*, and four in *Ae. vexans*, were observed. However, only four and one, respectively, contained significantly more observed than expected positive pools for *Cx. pipiens/restuans* and *Ae. vexans* (Table 3). One of the four significant *Cx. pipiens/restuans* clusters was over two months in the summer of the year 2012, and the other three clusters spanned the years 2015 to 2018 and temporally overlapped with each other (Table 3). Spatially, all four significant *Cx. pipiens/restuans* clusters were within and around the city of Ottawa, which is also where the highest numbers of positive pools per site were observed (Fig. 4C). The only significant *Ae. vexans* cluster spanned a few weeks in 2017 and was located around the city of Ottawa, which is where most positive pools were observed (Table 3; Fig. 4D).

**Figure 6:**
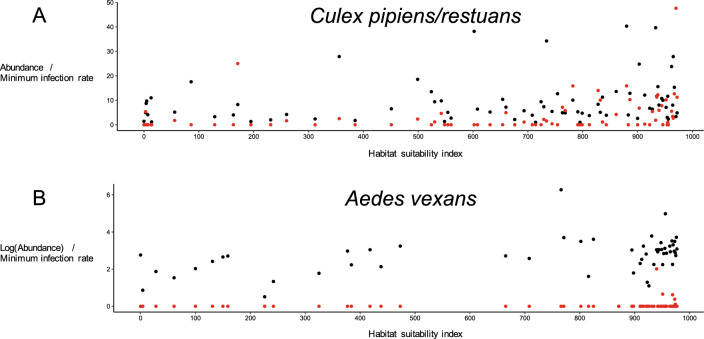
Scatter plots showing mean *Culex pipiens/restuans* **A** and *Aedes vexans* **B** abundance (black) and minimum infection rate (red) across sampling visits for each value of habitat suitability index. Abundance of *Aedes vexans* was log-transformed for easier visualization.

Mean HSI was higher within spatiotemporal clusters with significantly more observed than expected positive pools (at a threshold α of 0.05) both *Cx. pipiens/restuans* and *Ae. vexans*, compared to mean HSI calculated with respect to the species across the study area (Table 5). Across sampling sites, abundance and minimum infection rates seemed to increase as HSI increased in both species (Fig. 6).

**Table 5:** Mean and Confidence Interval (Mean ± 1.96 × Standard Deviation / √Cell Number) of Habitat Suitability Index Within Significant Detected Space–Time Clusters of High West Nile virus Rates for Both Study Species, *Culex pipiens/restuans* and *Aedes vexans*, Collected Across 118 Sampling Locations in Eastern Ontario, Canada, 2011–2020, and Across the Study Area.

Species	Mean habitat suitability index [confidence interval]	
	Within clusters	Across study area
*Cx. pipiens/restuans*	643 [635, 651]	322 [322, 322]
	761 [760, 762]	
	800 [796, 804]	
	559 [551, 567]	
*Ae. vexans*	897 [893, 901]	425 [425, 425]

## Discussion

This study’s objectives were to investigate spatiotemporal clusters of WNV-positive mosquito pools and the ecological niche of the two mosquito vector species/species groups, *Cx. pipiens/restuans* and *Aedes vexans*. We found spatiotemporal clusters of high WNV infection rates around the city of Ottawa in both mosquito vector species. We found highest habitat suitability for these same two principal vectors of WNV in the eastern half of the study area, where temperatures are generally warmer and residential and agricultural cropland cover is more dominant. Our study adds to previous results by contrasting the effect of residential land cover and vegetation cover, by including climatic variables, and by adding an analysis of *Ae. vexans*, which is also considered an important WNV vector species in southern Canada (Giordano et al. 2017; Anderson et al. 2020) and is generally associated with broader habitat suitability (Cloutier et al. 2021).

West Nile virus risk is often associated with suitable mosquito habitats within urban areas, such as urban wetlands and sewer overflow (Ruiz et al. 2007; Vazquez-Prokopec et al. 2010; Johnson et al. 2012). Spatiotemporal clustering analyses of West Nile virus infection in mosquitoes in North America identified proximity of rivers and water streams, high landscape fragmentation and density of roads as likely drivers of infection in mosquito pools, due to their association with WNV mosquito vector and avian reservoir abundance (Rochlin et al. 2011; Curtis et al. 2014; Myer et al. 2017). We therefore expected spatiotemporal WNV-positive mosquito pool clusters would be centered around the major urban population centers in our study area, such as found in a previous study in an area that was overlapping spatiotemporally with this study’s area, but spatially smaller, i.e., within the city limits of Ottawa, and temporally smaller and earlier in date, from 2007 to 2014. The two previously observed significant clusters included the year 2012 and were located in downtown Ottawa and one of its suburbs, and thus support one of the significant clusters detected in the study herein (Talbot et al. 2019a, 2019b). Our study’s three additional significant clusters for *Cx. pipiens/restuans* and one cluster for *Ae. vexans* occurred between 2015 and 2018, and are located in the same general area, i.e., in and around the city of Ottawa. Our results therefore add spatial and temporal depth to these previous results.

*Cx. pipiens/restuans* and *Ae. vexans* are associated with urban and/or man-modified landscapes, such as agricultural fields (Rocheleau et al. 2017; Giordano et al. 2018; Cloutier et al. 2021). Ecological niche of *Cx. pipiens/restuans* in a spatiotemporally overlapping area in Southeastern Ontario was previously found to be tightly linked to residential areas, namely in the large agglomerations of Ottawa and Toronto, and proximity to shrublands and forests were also associated with higher suitability (Moua et al. 2021). These results suggest that vegetated areas within urban landscapes were the most important land cover classes for *Cx. pipiens/restuans*. Our results support these earlier results, because we observed high habitat suitability for *Cx. pipiens/restuans* in residential areas with medium or high vegetation, but not with low vegetation cover. We observed similar associations for *Ae. vexans*. However, our results suggest areas well outside urban agglomerations, notably around agricultural croplands, are also highly suitable for both species. As in a study in Iowa (Larson et al. 2010), we found a strong association of *Ae. vexans* with open water and forested areas, and the suitability map was tightly linked to the two major rivers in our study area.

Previous results in Ontario did not account for climatic variables, which, according to our results, were responsible for the majority of the variation in habitat suitability index for both mosquito vector species. Temperature seemed to be limiting for both species below an annual average of 6°C and had a peak at an average of around 7°C, which may be explained by the tradeoff between optimal temperatures for host-seeking behavior, incubation and larval development (Di Pol et al. 2022). Precipitation was limiting for both species below an average of 2.5 mm and had a peak at an average of between 2.6 and 2.8 mm, which may be explained by the tradeoff between optimal rainfall for the creation of oviposition sites without flooding and flushing the larvae out of them (Chandra and Mukherjee 2022). However, variation in precipitation was low across the study area, so these effects would be best evaluated in a study area with more marked differences across sampling sites.

Our study contains one type of limitation, which concerns spatial aggregation of sampling sites around certain land cover classes, particularly around residential areas near the largest urban centers. However, the random forest algorithm and the Kulldorf’s spatial scan statistic perform well under these conditions. Therefore, we believe that most results from our niche modeling and spatiotemporal clustering analyses are reliable and meaningful.

Our study’s contributions include knowledge on the risk of West Nile virus near residential and agricultural areas demonstrated by ecological niche modeling and spatiotemporal clustering of positive mosquito pools of its two main mosquito vectors in Eastern Canada.

### Supplementary Information

Below is the link to the electronic supplementary material.

Description of each sampling site according to occurrence (1: >50%, 0:<50%), number of WNV-positive mosquito pools, geographic coordinates, land cover at the site and average daily total precipitations (mm) and average daily mean temperature (°C) at the site. (XLSX 17 KB)
